# Molecular evolution of the duplicated *TFIIAγ *genes in Oryzeae and its relatives

**DOI:** 10.1186/1471-2148-10-128

**Published:** 2010-05-04

**Authors:** Hong-Zheng Sun, Song Ge

**Affiliations:** 1 State Key Laboratory of Systematic and Evolutionary Botany, Institute of Botany, Chinese Academy of Sciences, Beijing 100093, China; 2 The Graduate University of the Chinese Academy of Sciences, Beijing 100039, China

## Abstract

**Background:**

Gene duplication provides raw genetic materials for evolutionary novelty and adaptation. The evolutionary fate of duplicated transcription factor genes is less studied although transcription factor gene plays important roles in many biological processes. TFIIAγ is a small subunit of TFIIA that is one of general transcription factors required by RNA polymerase II. Previous studies identified two *TFIIAγ*-like genes in rice genome and found that these genes either conferred resistance to rice bacterial blight or could be induced by pathogen invasion, raising the question as to their functional divergence and evolutionary fates after gene duplication.

**Results:**

We reconstructed the evolutionary history of the *TFIIAγ *genes from main lineages of angiosperms and demonstrated that two *TFIIAγ *genes (*TFIIAγ1 *and *TFIIAγ5*) arose from a whole genome duplication that happened in the common ancestor of grasses. Likelihood-based analyses with branch, codon, and branch-site models showed no evidence of positive selection but a signature of relaxed selective constraint after the *TFIIAγ *duplication. In particular, we found that the nonsynonymous/synonymous rate ratio (ω = *d*_N_/*d*_S_) of the *TFIIAγ1 *sequences was two times higher than that of *TFIIAγ5 *sequences, indicating highly asymmetric rates of protein evolution in rice tribe and its relatives, with an accelerated rate of *TFIIAγ1 *gene. Our expression data and EST database search further indicated that after whole genome duplication, the expression of *TFIIAγ1 *gene was significantly reduced while *TFIIAγ5 *remained constitutively expressed and maintained the ancestral role as a subunit of the TFIIA complex.

**Conclusion:**

The evolutionary fate of TFIIA*γ *duplicates is not consistent with the neofunctionalization model that predicts that one of the duplicated genes acquires a new function because of positive Darwinian selection. Instead, we suggest that subfunctionalization might be involved in *TFIIAγ *evolution in grasses. The fact that both *TFIIAγ1 *and *TFIIAγ5 *genes were effectively involved in response to biotic or abiotic factors might be explained by either Dykhuizen-Hartl effect or buffering hypothesis.

## Background

Transcription factors are large families in the genome of most eukaryotic organism and often act as switches between discrete developmental programs [1] and play important roles in many biological processes in plants, such as developmental regulation, control of metabolic pathways, response to environment stimuli and harmful stress [2,3]. Unlike regulatory transcription factors, general transcription factors are conserved proteins that are used by organisms as diverse as human, rat, *Drosophila*, and yeast to initiate mRNA synthesis [4]. TFIIA is one of general eukaryotic transcription factors required by RNA polymerase II and has been demonstrated to stimulate transcription by stabilizing TBP binding to the TATA box and by regulating TBP or TFIID dimerization to accelerate DNA binding [4,5]. All three polypeptides in TFIIA including the small subunits (TFIIA*γ*) showed high sequence and structural conservation across different organisms, highlighting their significance in eukaryotic transcription [6,7]. Recent studies showed that there were two *TFIIAγ*-like genes in rice genome, in contrast to *Arabidopsis *where only one copy was found [8]. Sequence comparison indicated that two rice *TFIIAγ*-like genes had 85.5% identity at the amino acid level and shared high degrees of nucleotide and amino acid sequence similarity with the *Arabidopsis TFIIAγ*-like gene [7,8]. Interestingly, a mutant (V39E substitution) in the copy on rice chromosome 5 (*xa5*) was confirmed to confer resistance to rice bacterial blight [7,8] and the other copy on chromosome 1 (*TFIIAγ1*) was found to be highly expressed when induced by pathogen invasion [9].

Gene duplication is widely recognized as a major evolutionary force shaping genome evolution, and provides raw genetic materials for evolutionary novelty and adaptation [10,11]. Duplication of transcription factor genes has been recently investigated, but almost all studies focused on regulatory transcription factors (e.g., [12-16]) and little is known about the evolution of basic transcription factor duplicates. The duplication and divergence of *TFIIAγ *gene in rice and their resistance reactions to rice bacterial blight raise a few of interesting questions. First, whether the new function of disease resistance is facilitated by the redundancy of *TFIIAγ *gene, as suggested by previous study [7]? Evidence showed that gene duplication might contribute to the ability of plants to obtain a defense response against disease and herbivory through the functional diversification of genes but empirical study is still scarce in plants [17,18]. Second, when the *TFIIAγ *duplication happened in history and what model fits the fate of the duplicated genes. The classic models of gene duplication predict that one of the duplicated genes is either lost by accumulation of deleterious mutations (pseudogenization or nonfunctionalization) [19,20] or acquires a new function because of positive Darwinian selection (neofunctionalization) [11,21]. Additional possible fates of the duplicated genes were also proposed, including maintenance of the ancestral function by both copies (redundancy) and subdivision of the ancestral function between copies (subfunctionalization and subneofunctionalization) [21-25]. Jiang et al. (2006) suggested that duplication of the *TFIIAγ *gene in rice gave rise to a new function for disease resistance during evolution. This hypothesis, however, remains to be justified by empirical molecular data. Molecular evolutionary analyses have been successfully used to test the alternative explanations for the retention and evolution of the duplicated genes (e.g., [14,16,26-29]). To reconstruct the phylogenetic relationships of *TFIIAγ *genes will help better elucidate the duplication history of two *TFIIAγ *and further reveal their evolutionary fates after the duplication.

Finally, we ask what role of selection plays on the evolution of duplicated *TFIIAγ *genes? Is there any change in the strength and mode of selection that have acted on the duplicate genes? What is the relative importance of relaxation of purifying selection and positive selection in the evolution of *TFIIAγ *genes? Previous studies often treated relaxation of purifying selection as the null hypothesis but positive selection after gene duplication has been well demonstrated (e.g., [28,30,31]). A few of current statistical methods provide effective ways to evaluate the role of positive selection following gene duplication and allow more specific cases can be addressed [28,32,33].

In the present study, we investigate the molecular evolution of the general transcription factor*TFIIAγ *in grasses, including a dense sampling of species of the rice tribe (Oryzeae). Based on the *TFIIAγ *gene phylogeny, we found that the duplication event giving rise to *TFIIAγ1 *and *TFIIAγ5 *happened in the common ancestor of extant grasses. Our molecular evolutionary analyses and likelihood ratio tests revealed the relaxation of selective constraint on *TFIIAγ *genes following gene duplication and an acceleration of *TFIIAγ1 *gene evolution. In conjunction with expression data, we demonstrated that both *TFIIAγ *genes following the duplication were functional and under strong selection constraint in Oryzeae and its relatives, providing no evidence that either gene evolved new functions or became a pseudogene despite their long-term coexistence for at least 50 MYA. Instead, the evolutionary fates of two *TFIIAγ *genes could be explained either by the Dykhuizen-Hartl effect [34] which predicts that one of duplicate genes evolves under relaxed purifying selection and later convey a selective advantage under particular environments, or by the buffering hypothesis which suggests that selection for a buffering effect is a mechanism for duplicate gene preservation after whole genome duplication.

## Methods

### Species samples

**Figure 1:**
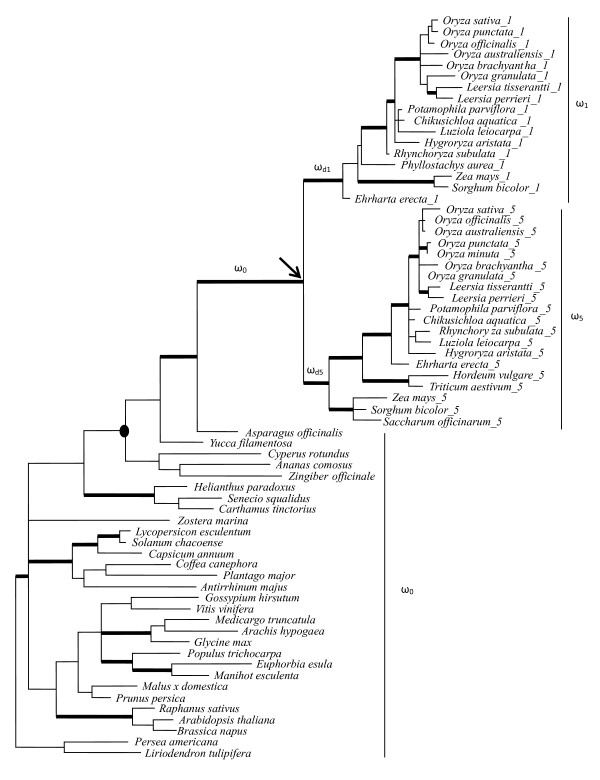
**Phylogeny of the *TFIIAγ*-like genes**. Phylogeny was inferred by Bayesian inference under the GTR+I+G model. Bold branches are supported by the Bayesian posterior probability > 0.90. Solid circle indicates the monocot group and the arrow indicates the duplication event. Sequences in bold were included in the pruned tree on which different branch models of molecular evolution were tested using the PAML analysis.

The rice tribe (Oryzeae) includes approximately 12 genera and more than 70 species distributed across the tropical and temperate regions of the world [35,36]. In this study, we sampled 13 diploid species that represent the main lineages of Oryzeae, including six *Oryza *species, two *Leersia *species, and one each of other five genera in the tribe (Figure 1; Additional file 1). One species in the tribe Ehrhartoideae that is sister to Oryzeae, *Ehrharta erecta*, was used as an outgroup [35,37]. To infer the duplication event of the two *TFIIAγ *genes, we selected additional 12 monocots and 24 dicots to generate the phylogenetic tree of the *TFIIAγ *genes. In total, 30 sequences were isolated here and the remaining sequences were extracted from GenBank by BLAST searches [38]. Detailed information of the species and the sequences and their GenBank accession numbers is listed in additional file 1.

### Isolation and sequencing of *TFIIAγ *genes

On the basis of the *TFIIAγ*-like sequences from rice, wheat and maize [Additional file 1], we designed two pairs of universal PCR primers to amplify the *TFIIAγ *genes. They are the forward primers P1 (5'-TTCgAgCTSTACMggMggTC-3') or P3 (5'-ATggCCACCTTCgAgCTSTA-3') and reverse primers P2 (5'-AggCCACRATCTTCACCTTg-3') or P4 (5'-TCRCAggCCACRATCTTCAC-3'). The regions amplified and the locations of the PCR and internal primers (P7 and P8) are shown in additional file 2. Genomic DNA was extracted from fresh young leaves or silica-gel dried leaves using the CTAB methods as described in [25]. PCR amplification was performed in a volume of 25 μl reaction using *ex*Taq polymerase (TaKaRa, Dalian, China). The cycling procedure was 35 cycles of denaturation at 94°C for 45 s, annealing at 56°C for 45 s and extension at 68°C for 8 min with 2 min of pre-denaturation and 10 min of final extension. PCR product was run on 1.2% agarose gel and all bands were excised under UV light, purified using Dinggou gel purification kit (Dingguo, Beijing, China), and sequenced using ET Terminator Kit (Amersham Pharmacia Biotech). All the PCR products were cloned into *pGEM *T-easy vectors (Promega, Madison, WI, USA) and at least 6 independent clones were sequenced. The purified fragments were also sequenced directly to make confirmation. If more than one copy was isolated in one species, we first construct a phylogeny including all the copies. If multiple copies from the same species clustered together, one copy was randomly selected in further analysis.

### Characterization of expression by RT-PCR and EST database search

Reverse transcriptase-polymerase chain reaction (RT-PCR) was performed to investigate whether there is difference of expression between two *TFIIAγ *genes. Total RNA was extracted from fresh leaves of eight species and young panicles of three species of Oryzeae [Additional file 1] using Plant RNA Reagent (Invitrogen, Carlsbad, California, USA). The first strand cDNA was reverse-transcribed with oligo dT_20 _primer. Subsequent detection was performed by PCR using up-stream primer P3 and low-stream specific primers P7 (5'-AYARWAACCTTgCTCTTgACTTgg-3') and P8 (5'-gACNNTAACCTTgCTCTTCACCTSA-3') (P7 for *TFIIAγ5 *copy and P8 for *TFIIAγ1 *copy). The *actin *gene was taken as control using primers ACT-59F (5'-AggCTggTTTCgCTggggATgATg-3') and ACTIN-764R (5'-ggACCTCggggCACCTgAACCTCT-3') [14]. The PCR procedure was 2 min of pre-denaturation at 94°C, 35 cycles of denaturation at 94°C for 30 sec, annealing at 54°C for 30 s, and extension at 72°C for 1 min, with a final extension of 10 min at 72°C. RT-PCR products were confirmed by sequencing.

In EST database search, all the hits of Poaceae species with e-value lower than 1e^-10 ^were collected. The sequences retrieved were aligned with rice *TFIIAγ1 *and *TFIIAγ5 *genes. By a neighbor-join phylogeny construction, all the sequences can be divided into two classes, corresponding to the *TFIIAγ1 *and *TFIIAγ5 *clades, respectively. We used the number of hits as an indicator of the expression level of the two copies, because a highly expressed gene would have greater chance to be picked from cDNA library than a lowly expressed gene [39,40].

### Sequence analysis

Sequences were aligned using a combination of methods implemented in BioEdit [41] and ClustalX 1.81 [42], with further manual refinements. The unalignable intron regions were excluded from the analyses. The GC content of all three codon positions and pairwise synonymous and nonsynonymous distances were calculated by MEGA3.1 [43]. Codon usage bias of the sequences was estimated by ENC (effective number of codons) that varies between 20 and 61, with the lower the value, the more biased codon usage [44]. We used Tajima's relative rate test [45], as implemented in MEGA3.1, to test for rate variation between two *TFIIAγ *genes using *Ananas comosus *as outgroup. To visualize conservation and check the rate variation along the *TFIIAγ *sequences, a sliding window analysis was performed by the K-estimator program [46]. Given relatively small length of *TFIIAγ *genes, we used a window size of 10 amino acid (30 bp) and a step size of 3 amino acid (9 bp) in the sliding window analysis. Poaceae species were used in the sliding analysis. To avoid sampling bias, only *Oryza punctata *was used to represent the Oryzeae species.

### Phylogenetic analysis

Phylogenetic analyses were performed using maximum likelihood (ML) method, implemented in PAUP 4.0b10 [47], and Bayesian inference (BI) with MrBayes v.3.12 [48]. For ML, heuristic searches were run with random taxon addition, tree bisection reconnection swap for 100 replications. The reliability of branches was evaluated by 500 bootstrap replications. In each bootstrap heuristic search replication, the same parameter settings were used, except that number of heuristic search replications was set to 10. In ML and analyses the best nucleotidfe substitution models for each data set were selected using Modeltest 3.7 by corrected Akaike information criterion [49]. In BI with GTR+I+G model, Markov chain Monte Carlo (MCMC) analysis was run for 1,000,000 generations, sampled every 100^th ^generations. The first 250,000 generations were set as burn-in.

We generated a phylogenetic tree of all *TFIIAγ *or *TFIIAγ*-like sequences to explore the duplication history of the two duplicates. For this purpose, we used only coding sequences to construct the gene tree because the intron sequences between *TFIIAγ1 *and *TFIIAγ5 *were unalignable. The phylogenetic tree was rooted by the *TFIIAγ*-like genes of *Liriodendron tulipifera *and *Persea americana *that belong to two families (Magnoliaceae and Lauraceae) of the basal angiosperms [50].

### Tests for selection

The ratio of nonsynonymous to synonymous substitution sites (*d*_N_/*d*_S _or ω) is an effective measure to detect selection on a gene or gene region [33]. If the ratio is significantly less than 1 (ω < 1), purifying selection is inferred, while positive selection is evoked if the ratio is significantly greater than 1 (ω > 1). An estimate of the ratio close to 1 (ω = 1) indicates the presence of neutral evolution. To explore the selective processes acting on *TFIIAγ *genes, we performed likelihood-based analyses using the codeml program of PAML version 4 [51]. We first tested whether the average ω ratio differed among lineages of the gene tree by using the branch models that allow ω to vary among lineages and assume different ω ratios assigned to the branches before and after the duplication event. The one ratio model (M0) assumes a single ω for all branches and all sites, whereas the other models allow for different ω ratios among branches of the tree. The free ratio model (Mf) assumes an independent ω ratio for each branch of the tree. The two ratio model M2r assumes one ω ratio to all branches predating the duplication event (ω_0_), and the other ratio to all branches postdating the duplication event (ω_d1 _= ω_d5 _= ω_1 _= ω_5_). The three ratio model (M3r) assumes one ratio restricted to all branches predating the gene duplication (ω_0_) and the other two to the branches of *TFIIAγ1 *(ω_d1 _= ω_1_) and *TFIIAγ5 *(ω_d5 _= ω_5_), respectively, following the duplication event. A more complex model, the four ratio model (M4r), assumes four independent ω ratios: one ratio restricted to all branches predating the gene duplication (ω_0_), one ratio to the branches immediately following the duplication (ω_d1 _= ω_d5_), and the last two assigned to the branches leading to *TFIIAγ1 *(ω_1_) and *TFIIAγ5 *(ω_5_) of grass species, respectively. Finally, the five ratio model (M5r) extends M4r to allow ω ratios to differ between the *TFIIAγ1 *and *TFIIAγ5 *branches immediately postdating the duplication (ω_d1 _≠ ω_d5_) (Figure 1; Table 1). A likelihood ratio test (LRT) was conducted to determine whether there is statistically significant difference between two models. If the LRT is significant, the null hypothesis that two models are not significantly different is rejected, and the model with higher likelihood value is assumed to be a better model [28,52].

**Table 1:** Log likelihood values and parameter estimates under different branch models and tests of hypotheses

Model	*p*	ln	Parameters for Branches	Models Compared	2ΔL
Mf: ω free	116	-4928.56	ω:0 ~ 0.513^a^	Mf vs. M0	184.74***
M0: ω_0 _= ω_d1_= ω_d5 _= ω_1 _= ω_5_	1	-5020.93	ω_0 _= ω_d1 _= ω_d5_= ω_1 _= ω_5 _= 0.055		
M2r: ω_0_≠ω_d1_= ω_1 _= ω_d5 _= ω_5_	2	-5015.74	ω_0 _= 0.046 ω_d1 _= ω_d5 _= ω_1_= ω_5 _= 0.077	M2r vs. M0	10.38**
M3r: ω_0_≠ω_d1_= ω_1_≠ω_d5 _= ω_5_	3	-5009.32	ω_0 _= 0.044 ω_d1 _= ω_1 _= 0.118 ω_d5 _= ω_5 _= 0.060	M3r vs. M2r	12.84***
M4r: ω_0_≠ω_d1_= ω_d5_≠ω_1_≠ω_5_	4	-5008.12	ω_0 _= 0.044 ω_d1 _= ω_d5 _= 0.085 ω_1 _= 0.122 ω_5 _= 0.066	M4r vs. M3r	2.40
M5r: ω_0_≠ω_d1_≠ ω_d5_≠ω_1_≠ω_5_	5	-5007.06	ω_0 _= 0.044 ω_d1 _= 0.043 ω_d5 _= ∞ ω_1 _= 0.121 ω_5 _= 0.066	M5r vs. M4r	2.12

^a ^zero *d*_*S *_branches are excluded.

**Significant at *P *< 0.01 level; *** Significant at the *P *< 0.001 level.

*p*, number of parameters.

We next used site-specific models to examine whether particular amino acid residues were subject to positive selection because the ω ratio is seldom detected greater than 1 if all the sites are averaged [53]. The nested codon models [28,54] were performed. In addition to one ratio model (M0), nearly neutral model (M1) classifies all the sites into 2 categories, one category under strict constraint (0 < ω < 1) and the other under neutral (ω = 1). Positive selection model (M2) is based on M1 and assumes a third category under positive selection (1 < ω). The discrete model (M3) classifies all the sites into several categories, each with a different ω ratio. Beta model (M7) assumes a beta distribution of the ω ratios, and beta&ω model (M8) extends an independent ratio estimated by the data. Models assuming positive selection M8 and M2 are compared with null models M7 and M1, respectively. Positive selection is invoked if the LRT is significant and there is site with ω > 1 [28]. A comparison between M3 and M0 can tell whether the ω ratio is homogeneous across different part of the gene.

We further performed the branch-site models A and B [55] to test for sites potentially under positive selection on *TFIIAγ1 *and *TFIIAγ5 *branches, respectively. Model A assumes 0 < ω_0 _< 1 and ω_1 _= 1 and was compared with nearly neutral model (M1); while model B determines ω_0 _and ω_1 _as free parameters to be estimated and compared with discrete model (M3) [55].

## Results

### Cloning and characterization of two *TFIIAγ *genes

Using genomic DNA we cloned and sequenced two *TFIIAγ *genes from all sampled Oryzeae species except for *Leersia tisserantti *for which only *TFIIAγ1 *was isolated, mainly because the second intron of *TFIIAγ5 *in this species was too long to be amplified successfully by *exTaq *DNA polymerase. However, when using cDNA template, we obtained the coding region of *TFIIAγ5 *and the first intron sequence using an internal primer for this species. Two *TFIIAγ *copies were also isolated and sequenced from other Poaceae species, including *Ehrharta erecta*, *Zea mays *and *Sorghum bicolor*. Only single *TFIIAγ*-like gene was isolated from both *Cyperus rotundus *and *Zingiber officinale *despite different attempts have been tried, including optimization of PCR amplification, recombination of up and down stream primers. All the *TFIIAγ *genes obtained in this study have three exons and two introns, with about 261 bp in coding sequence. The downloaded *TFIIAγ*-like sequences are cDNAs with full coding region. The *TFIIAγ1*-like sequences of rice, maize and sorghum were 327 bp in length and 9 bp (three codons) longer than the sequences of grass *TFIIAγ5*-like gene and those from the remaining species outside Poaceae. In Oryzeae, sequence length ranged from 1.3 to 1.8 kb for *TFIIAγ1 *and from 2.5 to 5.5 kb for *TFIIAγ5*. The first intron is about 70 ~100 bp in length for both genes, whereas the length of the second intron varied greatly [Additional file 2]. In coding regions, there is no indels between the two copies and can be aligned perfectly. We did not find the V39E substitution that lead to *TFIIAγ5 *(*xa5*) to confer resistance to rice bacterial blight in all Oryzeae species, indicating that such a mutation arises within *O. sativa*. The GC contents for the total and three individual codon positions were similar for the same gene but those at the 3rd position (GC_3_) is higher in *TFIIAγ1 *than in *TFIIAγ5 *(75.9% vs. 70.1%, *P *< 0.001) [Additional file 3]. Estimates of the codon usage showed that *TFIIAγ5 *had significantly lower ENC value than *TFIIAγ1 *(42.9 vs. 48.5, *P *< 0.001), paralleling its higher expression level in grasses (see below).

### Phylogeny of *TFIIAγ *genes

The alignment of all the coding sequences was 318 bp in length including gaps. Of them, 152 sites were parsimony informative. A Bayesian phylogeny indicated that all monocot species except for *Zostera marina *of Zosteraceae formed a monophyletic group, which forms polytomy with the other angiosperm clades. Such unsolved relationship reflects our current understanding of angiosperm phylogeny on which monocots were not resolved fully with many other basal angiosperms [50]. It is noted that all the *TFIIAγ *sequences from the Poaceae species formed two clades supported by Bayesian posterior probability > 90, one consisting of *TFIIAγ1 *homologs and the other *TFIIAγ5 *homologs (Figure 1). All the Oryzeae species and most grass species outside Oryzeae have two distinct types of *TFIIAγ *sequences that fell into the two clades. In some grass species, only one *TFIIAγ*-like sequence was isolated, which formed a cluster with either *TFIIAγ1*-like or *TFIIAγ5*-like clade. In contrast, a single *TFIIAγ*-like copy was found in two species from the families closely related to Poaceae, *Cyperus rotundus *of Cyperaceae and *Zingiber officinale *of Zingiberaceae. Moreover, the monocot clade is sister to the *TFIIAγ*-like sequences from the remaining angiosperm species (Figure 1). ML analyses produced similar tree topologies [Additional file 4]. These observations indicated that the duplication event giving rise to *TFIIAγ1 *and *TFIIAγ5 *occurred at the ancestors of Poaceae or before the divergence of Poaceae.

### Sequence conservation and rate difference between two *TFIIAγ *genes

**Figure 2:**
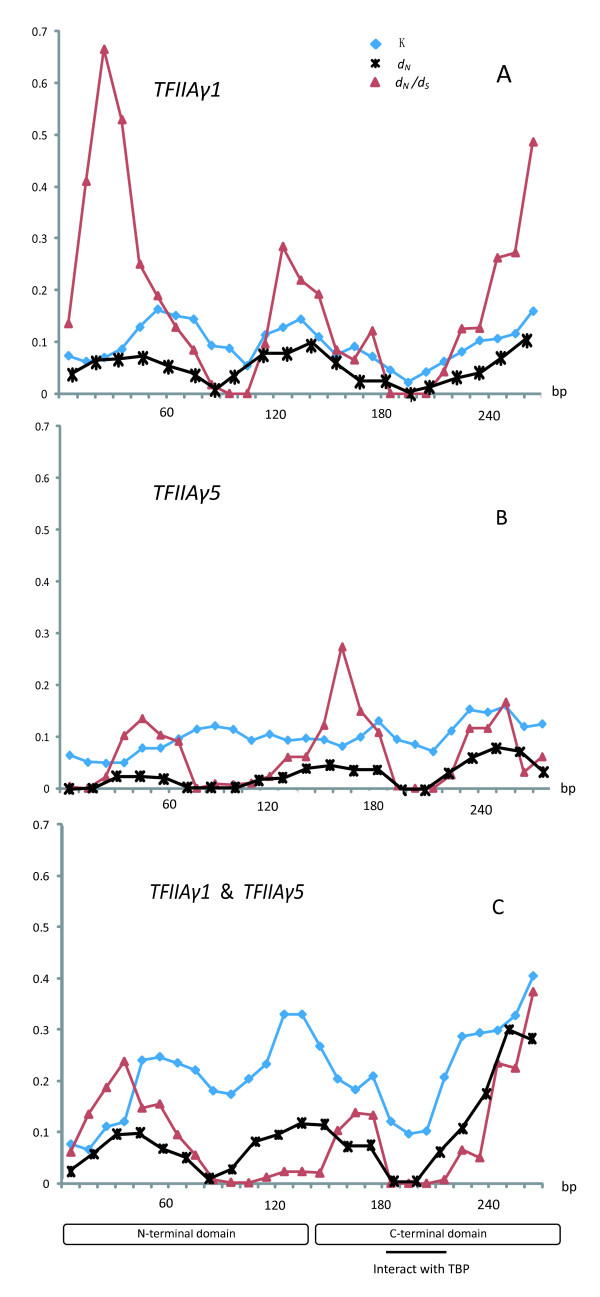
**Sliding window analysis of *TFIIAγ*-like sequences**. Sliding window plots of average nucleotide divergence (*K*), *d*_*N *_and *d*_*N*_/*d*_*S *_were made for all pairwise comparisons between (A) *TFIIAγ1 *orthologs, (B) *TFIIAγ5 *orthologs and (C) the paralogs of *TFIIAγ1 *and *TFIIAγ5*. Two functional domains and the sites that interact with TBP are shown in boxes and bar, respectively. Window size = 30 bp and step size = 9 bp.

We performed a sliding window analysis by calculating the nucleotide divergence of the entire sequence with JC model (K), of nonsynonymous (*d*_*N*_) and synonymous substitution sites (*d*_*S*_). The *d*_*N *_values for both genes were lower than those of *d*_*S *_(*d*_*N*_/*d*_*S*_≤ 0) in almost all sliding windows but all three parameters fluctuated across the genes (Figure 2). The conserved regions in *TFIIAy1 *are different from those in *TFIIAy5 *and some sites in *TFIIAγ1 *might experience relaxation of selective constraints with elevated *d*_*N*_/*d*_*S *_values relative to those of *TFIIAγ5 *(Figure 2A and 2B). In addition, both the K and *d*_*N *_values of *TFIIAγ1 *were higher than those of *TFIIAγ5*, suggesting higher rate of evolution in *TFIIAγ1 *genes. To detect the potential impact of intergenic conversion on molecular evolution [56], we further calculated the parameters between two paralogs (Figure 2C). We did not find significant difference in evolutionary rates between two domains in which heterogeneity occurred across the sequences. It is evidence that low sequence differentiation was found around the functional regions (e.g., the region that interact with TBP), inconsistent with variation pattern of gene conversion that sequence divergence would occurred around the functional site [56].

Relative rate test was used to compare the *TFIIAγ1 *and *TFIIAγ5 *sequences from the main lineages in grasses in relations to the *TFIIAγ*-like sequence from *Ananas comosus *of the family Bromeliaceae that is closely related to Poaceae [50]. For all paralogs from 12 species tested, *TFIIAγ1 *evolved 1.14 to 1.34 times faster than *TFIIAγ5 *(Table 2). The tests were statistically significant or marginal significant for six out of 12 species. When more distinctly related species *Zingiber officinale *was used as an outgroup, the results were similar in that *TFIIAγ1 *evolved faster than *TFIIAγ5 *in all 12 species though the tests were not significant (Table 2). We calculated the synonymous and nonsynonymous substitution rates of *TFIIAγ1 *and *TFIIAγ5 *between the Oryzeae species and found that the average *d*_*N *_value of *TFIIAγ1 *was significantly higher than that of *TFIIAγ5 *(0.033 vs. 0.011, *P *< 0.001); the pairwise *d*_*S *_values of *TFIIAγ1 *and *TFIIAγ5 *were also significant (0.155 vs. 0.131, *P *= 0.001). The accelerated *d*_*N *_in *TFIIAγ1 *is obvious when we examined the amino acid alignments for the two genes, in which 21 sites had amino acid mutations in *TFIIAγ1 *in contrast to 14 sites in *TFIIAγ5 *[Additional file 5]. The overall ω (*d*_*N*_/*d*_*S*_) values for both genes were far below 1 (0.213 for *TFIIAγ1 *and 0.084 for *TFIIAγ5*), indicating both genes were subjected to selection constraint, but the constraint on *TFIIAγ5 *was stronger.

**Table 2:** Tajima's relative rate tests for *TFIIAγ1*/*TFIIAγ5 *duplicates using *Ananas comosus *and *Zingiber officinale *as outgroups

Species	*Ananas comosus*			*Zingiber officinale*		
	γ1/γ5^a^	χ^2^	*P*-value	γ1/γ5	χ^2^	*P*-value
*Oryza sativa*	1.14	1.32	0.250	1.18	1.60	0.206
*Oryza brachyantha*	1.19	2.31	0.128	1.09	0.42	0.516
*Oryza granulata*	1.14	1.26	0.262	1.07	0.23	0.631
*Leersia tisserantti*	1.14	1.09	0.297	1.07	0.20	0.655
*Potamophila parviflora*	1.29	4.83	0.028*	1.16	1.26	0.262
*Chikusichloa aquatica*	1.26	4.00	0.046*	1.11	0.64	0.423
*Rhynchoryza subulata*	1.21	2.78	0.096	1.14	0.95	0.330
*Hygroryza aristata*	1.20	5.77	0.016*	1.19	1.68	0.194
*Luziola leiocarpa*	1.34	2.19	0.139	1.19	1.52	0.217
*Ehrharta erecta*	1.22	3.57	0.059	1.20	2.61	0.106
*Zea mays*	1.27	4.33	0.037*	1.21	2.94	0.086
*Sorghum bicolor*	1.15	2.13	0.144	1.05	0.29	0.590

^a ^The ratio of the genetic distance (Kimura 2-parameter) between *TFIIAγ1 *and the outgroup over that between *TFIIAγ5 *and the outgroup.

* indicates significance at *P *< 0.05 level.

### Selection constraints among lineages

We used different kinds of likelihood ratio tests to examine whether there was variation of ω ratios on different lineages and, in particular, whether there is any increase in the ω ratio after the *TFIIAγ *duplication. Free ratio (Mf) and two ratio (M2r) models both have significantly higher likelihood scores than one ratio model (M0), rejecting the null hypothesis that the *TFIIAγ*-like genes have evolved at constant rates along branches (Table 1). However, branch-specific ω values under Mf model were all lower than one (ranging from 0 ~0.513), suggesting that purifying selection or constraint on amino acid sequence best explains the evolution of *TFIIAγ*-like genes in angiosperms. Two ratio model, with ω_0 _= 0.046 for all branches before the *TFIIAγ *duplication and ω_d1 _= ω_d5 _= ω_1 _= ω_5 _= 0.077 for the branches after the duplication, fits the data significantly better than one ratio models (M2r vs. M0, 2ΔL = 10.38, *P *< 0.001), indicative of a significant increase in ω value following the duplication event. We further calculated the likelihood under comparison between models M3r and M2r to explore the assumption of the same selective constraints at two *TFIIAγ *genes after the duplication event. Likelihood of model M3r was significantly better than M2r (2ΔL = 12.84, *P *< 0.0001), suggesting that different selective pressures occur in the two *TFIIAγ *genes with stronger purifying selection in *TFIIAγ5 *(ω_d5 _= ω_5 _= 0.060) than in *TFIIAγ1 *gene (ω_d1 _= ω_1 _= 0.118). Finally, the comparison between M5r and M4r indicated that the ω ratios of the two branches immediately following the duplication event were not significantly different from each other (2ΔL = 2.12, *P *= 0.145) (Table 1), implying that the asymmetric rates of *TFIIAγ *evolution occurred mainly after diversification of grasses.

### Detecting positive selection in *TFIIAγ *genes

Given the fact that the selective constraints on *TFIIAγ *genes relaxed after duplication and conferred disease resistant or induced by pathogen in cultivated rice, it is interesting to ask whether any accelerated rate of relaxation happen and any amino acid residue is potentially under positive selection. Because the branch model test averages the ω ratios across all sites and is a very conservative test of positive selection [33], we applied site-specific and branch-site models to *TFIIAγ *dataset. As shown in Table 3, site-specific modelsindicate that *TFIIAγ *genes were under strong purifying selection with ω = 0.055 in one-ratio model (M0). The discrete model (M3) was significantly better than M0 (2ΔL = 193.62, *P *< 0.001), indicating that the ω ratio was not homogeneous among sites along the sequence. This is also obvious in the sliding window analysis (Figure 2) and the amino acid alignment of *TFIIAγ *genes [Additional file 5]. Models M2 and M8 assuming positive selection were not significantly better than the null models M1 and M7 (for M1 vs. M2, 2ΔL = 0.0, *P *= 1.0; for M7 vs. M8, 2ΔL = 0.0, *P *= 1.0), and no site was found to be under positive selection by Bayes Empirical Bayes (BEB) inference [32] using a probability criterion of 95%. Thus, the nearly neutral model was better to explain the data. In model M1, about 94% of the codons are under strict constraint (ω = 0.030), and the other 6% codons are under neutral evolution (ω = 1.0) (Table 3).

**Table 3:** Parameters and likelihood scores of *TFIIAγ *genes under codon and branch-site models

Model	*p*	ln	2ΔL	Estimate of parameters	Positively selective site
M0: one ratio	1	-5020.93	184.74**	ω:0.055	none
Codon model					
M1: nearly neutral	1	-4915.09	211.68**	p_0 _= 0.938, p_1 _= 0.062 ω_0 _= 0.038, ω_1 _= 1.0	not allowed
M2: positive selection	3	-4915.09	0	p_0 _= 0.938, p_1 _= 0.044, p_2 _= 0.017 ω_0 _= 0.038, ω_1 _= 1.0, ω_2 _= 1.0	none
M3: discrete	5	-4818.28	193.62**	p_0 _= 0.714, p_1 _= 0.224, p_2 _= 0.063 ω_0 _= 0.008, ω_1 _= 0.124, ω_2 _= 0.480	none
M7: beta	2	-4815.29		p = 0.200, q = 2.073	not allowed
M8: beta & ω	4	-4815.29	0	p_0 _= 1.0, p = 0.200, q = 2.073 p_1 _= 0, ω = 2.0	none
Branch-site model					
Foreground: *TFIIAγ1*					
Model A	3	-4915.04	0.10	p_0 _= 0.910, p_1 _= 0.060 (p_2_+p_3 _= 0.030) ω_2 _= 1.0	none
Model B	5	-4840.74	-44.92	p_0 _= 0.806, p_1 _= 0.194 (p_2_+p_3 _= 0) ω_0 _= 0.015,ω_1 _= 0.259, ω_2 _= 0	none
Foreground: *TFIIAγ5*					
Model A	3	-4908.26	13.66**	p_0 _= 0.929, p_1 _= 0.058 (p_2_+p_3 _= 0.013) ω_2 _= ∞	90T
Model B	5	-4840.74	-44.92	p_0 _= 0.806, p_1 _= 0.194 (p_2_+p_3 _= 0) ω_0 _= 0.015, ω_1 _= 0.259, ω_2 _= 0	none

**Significant at *P *< 0.01 level; *** Significant at the *P *< 0.001 level.

*p*, number of parameters.

We further tested for evidence of positive selection on two *TFIIAγ *genes separately using branch-site models (Table 3). Branch-site models A and B specifying branch *TFIIAγ1 *as the foreground branch were not significantly better than the null models M1 (2ΔL = 0.1, *P *= 0.95) and M3 (2ΔL = -44.92, *P *= 1.0). In analyses of the branch *TFIIAγ5*, however, model A was significantly better than the null model (2ΔL = 13.66, *P *< 0.001) with ω ratio greater than 1, but model B was not significantly better than the null model (Table 3). We checked the inferred positive selection site (90T) across all protein sequences and found that it was fixed in both copies, with all *TFIIAγ1 *genes being T and *TFIIAγ5 *genes Q [Additional file 5]. This observation suggests it unlikely that positive selection occurs in either copy in grasses. Alternatively, this site might experience positive selection immediately after duplication of *TFIIAγ *gene in ancestor of grasses and then fixed under strong purifying selection in grasses. It should be noted that the *TFIIAγ5 *gene was highly expressed with significantly lower ENC relative to *TFIIAγ1 *gene [Additional file 3]. Therefore, the ω value greater than one at 90 site of *TFIIAγ5 *gene might be caused by low *d*_*S *_value rather than positive selection because synonymous sites are likely to be under negative selection in highly expressed genes due to codon usage bias [57].

### Gene expression of the *TFIIAγ *genes

**Figure 3:**
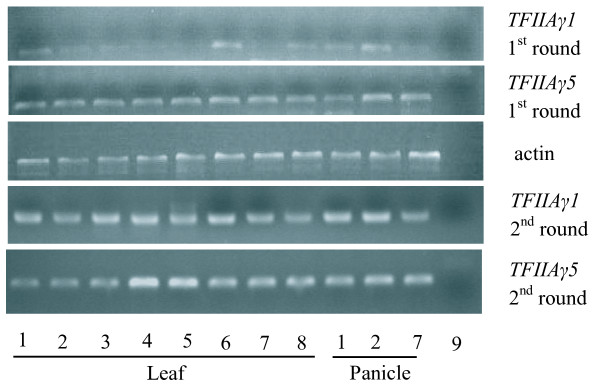
**RT-PCR results**. 1. *Oryza sativa*; 2. *Oryza punctata*; 3. *Oryza officinalis*; 4. *Oryza australiensis*; 5. *Leersia tisserantti*; 6. *Hygroryza aristata*; 7. *Zizania latifolia*; 8. *Rynchoryza subulata*; 9. Blank control.

Two rounds of RT-PCR were performed to determine the expression of *TFIIAγ1 *and *TFIIAγ5 *genes in tribe Oryzeae species. In the first round, equal amount of template cDNA was added in the reaction of *TFIIAγ1 *and *TFIIAγ5*. The expression of *TFIIAγ5 *was detected in all the leaves and young panicles, while the expression of *TFIIAγ1 *was weaker than that of *TFIIAγ5 *for most expected bands, and were almost invisible in *O. officinalis, O. australiensis *and *Leersia tisserantti *(Figure 3). The weaker bands of *TFIIAγ1 *indicated that it was expressed at lower level relative to *TFIIAγ5*. When a second round PCR was taken, the expected bands appeared in all the species. To avoid contamination, all RT-PCR products of *TFIIAγ1 *and *TFIIAγ5 *were confirmed by sequencing, and the resulting sequences were identical to the coding regions of genomic sequences in each species. These results showed that both copies were expressed in leaf and young panicle of Oryzeae species, but the *TFIIAγ5 *was expressed at higher level.

Different expression levels of two *TFIIAγ *genes were further confirmed by the GenBank EST database search using rice *TFIIAγ1 *and *TFIIAγ5 *sequences. Both copies were found in rice, maize and sorghum, but the hits of *TFIIAγ5 *far outnumbered those of the *TFIIAγ1 *copy in rice and maize [Additional file 6]. In several other Poaceae species, only the *TFIIAγ5 *copy was found. The low number of hits indicated that the *TFIIAγ1 *expression was much lower than that of *TFIIAγ5*, consistent with our RT-PCR findings. In addition, the matches of *TFIIAγ5 *expression were found in all types of cDNA libraries, including the callus, mature or immature tissue, stressed or unstressed and different developing stage libraries; whereas the *TFIIAγ1 *hits appeared mainly in drought-stressed tissue, pollen, immature and meristematic and mixed libraries [Additional file 6]. These observations suggest that *TFIIAγ5 *might be constitutively expressed and *TFIIAγ1 *be expressed under stress induction or expressed in specific tissues.

## Discussion

This study identified two *TFIIAγ *genes for all Oryzeae species and the representatives of grass species, which formed two monophyletic clades corresponding to the rice *TFIIAγ1 *and *TFIIAγ5 *genes; whereas only a single copy was found for the remaining monocots and angiosperm species. Phylogenetic analyses of all the *TFIIAγ*-like sequences indicated that the duplication of *TFIIAγ *into *TFIIAγ1 *and *TFIIAγ5 *occurred before the divergence of rice and maize (Figure 1). This implies that the duplication event that gave rise to *TFIIAγ1 *and *TFIIAγ5 *genes might occur before the common ancestor of extant grasses because rice (subfamily Ehrhartoideae) and maize (subfamily Panicoideae) are two distinctly related lineages in the grass family [58,59].

It has been demonstrated that the rice genome experienced two large-scale duplications, one whole genome duplication occurred about 70 MYA, and an additional segmental duplication happened 5 ~ 21 MYA involving chromosomes 11 and 12 [60-62]. Previous studies found that the location of two rice *TFIIAγ *genes corresponded to a large-scale duplication of a portion of rice chromosomes 1 and 5 [7,8]. To determine whether the timing of the duplication event leading to *TFIIAγ1*/*TFIIAγ5 *is consistent with the whole genome duplication around 70 MYA, we calculated the synonymous distance (*d*_*S*_) between *TFIIAγ *orthologs and paralogs for rice and maize by the method of Nei and Gojobori (1986). The *d*_*S *_distances between the *TFIIAγ *orthologs were 0.388 for rice and 0.457 for maize and those between the paralogs of rice and maize were 0.592 (*TFIIAγ1*) and 0.497 (*TFIIAγ5*), respectively. According to a molecular clock assuming rice and maize diverged 50 MYA [58], the *TFIIAγ1 *and *TFIIAγ5 *paralogs diverged about 54 ~76 MYA. This date coincides with the time scale that Poaceae diverged 55 ~ 77 MYA [58,59]. Wang et al. (2005) identified 10 large duplicated blocks arising from the whole genome duplication, including two blocks involving chromosomes 1 and 5. Our further search on rice genome found that two rice *TFIIAγ *genes located on block 10 determined by Wang et al. (2005). Therefore, the *TFIIAγ *duplication is within a large duplicated segment of rice genome and most likely to arise following a whole genome duplication event that was assumed to have occurred before the divergence of Poaceae [60-62].

Our timing of the *TFIIAγ *duplication suggests that the *TFIIAγ1 *and *TFIIAγ5 *paralogs have been maintained in the grass genome for a considerable amount of time (at least 50 MYA). This implicates that selection rather than random drift is responsible for the retention of both *TFIIAγ *activities during grass evolution because most gene duplicates have a short lifespan (within a few million years after duplication) before one copy was deleted (pseudogenization) [24]. It has been well established that gene duplication is often followed by an elevated rate of protein evolution and a large proportion of the duplicate pairs displayed asymmetric evolution, i.e., one of the duplicates evolves much faster than the other [19,29,63-65]. Conant and Wagner (2003) analyzed four completely sequenced genomes and found that 20% - 30% of duplicate gene pairs showed asymmetric evolution in the amino acid sequence, and particularly, the greater this asymmetry, the greater the *d*_N_/*d*_S _ratio in a gene pair, indicating that most asymmetric divergence might be caused by relaxed selective constraints on one of the duplicates. In well agreement with previous studies, we found significantly higher ω ratios for branches arising from the duplication event in rice tribe and its relatives, suggesting weaker purifying selection on the duplicate genes during diversification of grasses after the duplication event. Moreover, the ω ratios of the *TFIIAγ1 *sequences are two times higher than those of *TFIIAγ5 *sequences, consistent with the results of relative rate tests in which *TFIIAγ1 *evolved faster than *TFIIAγ5 *(Table 2). Such an asymmetric evolution of the *TFIIAγ *duplicates reflects an acceleration of evolutionary rate of *TFIIAγ1 *relative to *TFIIAγ5*. Our likelihood-based analyses with both branch and codon models showed no evidence of positive selection but a signature of relaxed selective constraint after the *TFIIAγ *duplication and subsequent acceleration of *TFIIAγ1 *gene. The low ω values (0.060 ~ 0.118) across the branches leading to both *TFIIAγ *duplicates also suggest that strong selection constrains remain for the two copies after the duplication, with *TFIIAγ1 *evolving under weaker selective constraint in grass species.

The fate of duplicated genes has been a hot debate since Ohno (1970), and several hypotheses have been proposed to interpret the preservation of both copies, including neofunctionalization [11], subfunctionalization [21,24], subneofunctionalization [66] and some other models (see review in Semon and Wolfe 2007). Based on sequence analyses and expression data, Iyer and McCouch (2004) found that the recessive mutation on *TFIIAγ5 *locus for resistance to rice bacterial blight did not affect the essential function of *TFIIAγ *gene and hypothesized that *TFIIAγ5 *functioned both as a general transcription factor and as a resistance gene (*xa5*) in rice, which was further demonstrated by subsequent complementation test and 3-D structure prediction [7]. We conducted a secondary structure prediction of the *TFIIAγ1 *and *TFIIAγ5 *proteins of grass species and found little difference in the secondary structures between the two copies [Additional file 5]. These observations, in combination of our molecular evolutionary analyses (Tables 1 and 3), demonstrated that both *TFIIAγ *genes were functional and under selection constraint in Oryzeae and its relatives. Thus, pseudogenization is unlikely involved in *TFIIAγ *evolution. Because extra amounts of protein or RNA products such as rRNAs and histones are in high demand [22], the retention of both *TFIIAγ *copies might be attributed partly to the importance of *TFIIAγ *as a component of TFIIA that is a general transcription factor needed in all polymerase II transcriptions [4,5].

Jiang et al (2006) investigated the expression patterns of two *TFIIAγ *genes in rice and indicated that the *TFIIAγ1 *gene was not expressed in young panicle, in contrast to *TFIIAγ5 *that expressed in all organs tested (leaf, stem, panicle, and root). In our study on *O. sativa*, *O. punctata *and *Z. latifolia*, however, the expression of *TFIIAγ1 *was detected in both leaves and young panicles but the expression level was much lower relative to *TFIIAγ5 *gene (Figure 3). These observations, in conjunction with our expression data, indicate that after whole genome duplication, the expression of *TFIIAγ1 *copy was significantly reduced while *TFIIAγ5 *remained constitutively expressed and maintained the ancestral role as a subunit of the TFIIA complex. Consequently, it seems that subfunctionalization might be involved in *TFIIAγ *evolution in grasses. The case of *TFIIAγ *genes agree with previous notion that subfunctionalization would lead to functional specialization when one of the duplicate genes became better at performing the original function of the progenitor gene [22]. Nevertheless, the possibility that positive selection on some specific sites immediately after duplication of *TFIIAγ *gene in ancestor of grasses cannot be excluded entirely given short length of the *TFIIAγ *gene and the inference power of methods in our case [67].

One important point for the evolution of *TFIIAγ *genes is the evidence that both *TFIIAγ1 *and *TFIIAγ5 *genes were effectively involved in response to biotic or abiotic factors. In addition to *xa5 *mutation that lead to resistance to rice bacterial blight, a recent study documented that the expression of *TFIIAγ1 *could express 400-fold greater than normal when infected by specific bacterial races (PXO99^A^) that cause blight disease [9]. Our EST database search also found the frequent presence of *TFIIAγ1 *gene in drought-stressed cDNA library both in rice and sorghum, implying its inducibility by drought stress [Additional file 6]. As pointed out by previous authors, gene redundancy might create subtle fitness advantage that was only evident in particular stages of the life cycle or under particular environments [25,68,69]. Therefore, the fate of the duplicated *TFIIAγ *genes can be alternatively explained by the Dykhuizen-Hartl effect [31,34], which predicts that one of duplicate genes evolves under relaxed purifying selection and the fixed mutations later convey a selective advantage in a novel environment or genetic background. It is noted that the V39E substitution in the α-helix domain of *TFIIAγ5 *was confined only to some varieties of *O. sativa*, suggestive of its recent emergence [7,8] [Additional file 5].

The involvement of the duplicated *TFIIAγ *genes in adversity response could also be explained by the buffering hypothesis [27], which suggests that selection for a buffering effect was a mechanism for duplicate gene preservation after whole genome duplication. By exploring the footprints of selection associated with genome duplication in *Arabidopisis *ecotypes and rice subspecies, Chapman et al. (2006) found that functional buffering might be important against genetic turbulence after genome duplication and could continue to act ~60 million years later. Retention of duplicate genes, particularly for complex genes and gene network, plays a critical role for genetic robustness of biological systems [22,25,27,70,71]. TFIIA is a complex consisting of three polypeptides and assumed recently to be tightly regulated with a particular role in differentiation and development [6]. Further biochemical and molecular investigations on the respective functions and the interactions between TFIIAγ and the other two components will be required to better understanding of the biology of the transcription factor TFIIA and to provide useful insights into the evolution of *TFIIAγ *and its counterparts.

## Conclusions

Based on phylogenetic reconstruction of the *TFIIAγ *genes from main lineages of angiosperms, we demonstrated that two *TFIIAγ *genes (*TFIIAγ1 *and *TFIIAγ5*) arose from a whole genome duplication that happened in the common ancestor of grasses. Likelihood-based analyses with different models showed no evidence of positive selection but a signature of relaxed selective constraint after the *TFIIAγ *duplication. In particular, the nonsynonymous/synonymous rate ratio (ω = *d*_N_/*d*_S_) of the *TFIIAγ1 *sequences was two times higher than that of *TFIIAγ5 *sequences, indicating highly asymmetric rates of protein evolution in rice tribe and its relatives. Our expression data and EST database search further indicated that after whole genome duplication, the expression of *TFIIAγ1 *gene was significantly reduced while *TFIIAγ5 *remained constitutively expressed and maintained the ancestral role as a subunit of the TFIIA complex. These observations are not consistent with the neofunctionalization model that predicts that one of the duplicated genes acquires a new function and instead, implicate that subfunctionalization might be involved in *TFIIAγ *evolution in grasses. The fact that both *TFIIAγ1 *and *TFIIAγ5 *genes were effectively involved in response to biotic or abiotic factors might be explained by either Dykhuizen-Hartl effect or buffering hypothesis.

## Abbreviations

TBP: TATA-binding protein; ENC: effective number of codons; EST: expressed sequence tags; ML: maximum likelihood; BI: Bayesian inference; MCMC: Markov chain Monte Carlo.

## Authors' contributions

SG and HZS designed the research and outlined the manuscript together. HZS performed the research. HZS and SG analyzed and interpreted the data. SG and HZS wrote the paper. Both authors have read and approved the final manuscript.

## Supplementary Material

Additional file 1 *TFIIAγ*-**like sequences included in this study.** Click here for file

Additional file 2 **Gene structure and the location of primers. **Universal forward (P1 and P3) and reverse (P2 and P4) primers are shown above the genes and the copy-specific internal sequencing primers (P7 and P8) are shown below the gene. Exons are shown in boxes and the shaded boxes are coding regions. Click here for file

Additional file 3 GC contents (%) and ENC of *TFIIAγ1 *and *TFIIAγ5 *in Oryzeae species and its relative. Click here for file

Additional file 4 Maximum likelihood tree using GTR+I +G model of evolution. Bootstrap values > 50% are shown above branches. Click here for file

Additional file 5 **Amino acid alignment of the *TFIIAγ *genes.** 2D structure in the bottom is predicted by PredictProtein http://www.predictprotein.org/ using *O. sativa *sequences as references. H represents the alpha helix and E the beta strand. Click here for file

Additional file 6 EST hits of grass *TFIIAγ *genes in GenBank EST database. Click here for file
