# Supplementary material for: Molecular evolution of the duplicated TFIIAγ genes in Oryzeae and its relatives
Source: BMC Evol Biol. 2010 May 4;10:128. doi: 10.1186/1471-2148-10-128 (PMC2887407; doi:10.1186/1471-2148-10-128)
Supplement: Additional file 1 — TFIIAγ-like sequences included in this study. [file 1471-2148-10-128-S1.PDF]

TFIIAγ-like sequences included in this study

Taxon	Order / Family	Origin / Accession No.[b]	GenBank Accession No. [c]
Oryza minuta	Poales / Poaceae	—	CB210904
Oryza sativa [a]	Poales / Poaceae	—	**EF577601, EF577594**
Oryza punctata [a]	Poales / Poaceae	Cameroon / **105984**	**EF577602, EF577595**
Oryza officinalis [a]	Poales / Poaceae	Bangladesh / **102460**	**EF577603, EF577596**
Oryza astraliensis [a]	Poales / Poaceae	Australia / **101410**	**EF577604, EF577597**
Oryza brachyantha	Poales / Poaceae	Sierra Leone / **105151**	**EF577605, EF577598**
Oryza granulata	Poales / Poaceae	Vietnam / **106469**	**EF577606, EF577599**
Leersia tisserantti [a]	Poales / Poaceae	Guinea / **101384**	**EF577607, EF577600**
Leersia perri	Poales / Poaceae	Madagascar / **105164**	**EU583805, EU583814**
Potamophila parviflora	Poales / Poaceae	Australia / **85424**	**EU583806, EU583815**
Chikusichloa aquatica	Poales / Poaceae	Japan / **106186**	**EU583807, EU583816**
Rhynchoryza subulata [a]	Poales / Poaceae	Argentina / **100913**	**EU583808, EU583817**
Luziola leiocarpa	Poales / Poaceae	Argentina / **82043**	**EU583809, EU583818**
Hygroryza aristata [a]	Poales / Poaceae	China / JXHA01	**EU583810, EU583819**
Ehrharta erecta	Poales / Poaceae	U.S.A. / 4698	**EU583811, EU583820**
Zizannia latifolia [a]	Poales / Poaceae	China / BJB7	—
Zea mays	Poales / Poaceae	—	CA826708, BG836616
Sorghum bicolor	Poales / Poaceae	—	CX622871, CF483781
Phyllostachys aurea	Poales / Poaceae	—	**EU583812**
Triticum aestivum	Poales / Poaceae	—	BT008987
Hordeum vulgare	Poales / Poaceae	—	CA022917
Saccharum officinarum	Poales / Poaceae	—	CA260612

Ananas comosus	Poales / Bromeliaceae	—	DT339451
Cyperus rotundus	Poales / Cyperaceae	China / HNCR01	**EU583821**
Zingiber officinale	Zingiberales / Zingiberaceae	—	DY365620
Asparagus officinalis	Asparagales / Asparagaceae	—	CV288112
Yucca filamentosa	Asparagales / Agavaceae	—	DT595413
Zostera marina	Alismatales / Zosteraceae	—	AM766131
Helianthus paradoxus	Asterales / Asteraceae	—	EL490508
Senecio squalidus	Asterales / Asteraceae	—	DY664226
Carthamus tinctorius	Asterales / Asteraceae	—	EL408922
Lycopersicon esculentum	Solanales / Solanaceae	—	BP905110
Capsicum annuum	Solanales / Solanaceae	—	CO910585
Solanum chacoense	Solanales / Solanaceae	—	DN983032
Coffea canephora	Gentianales / Rubiaceae	—	DV682628
Plantago major	Lamiales / Plantaginaceae	—	AM111318
Antirrhinum majus	Lamiales / Plantaginaceae	—	AJ558785
Gossypium hirsutum	Malvales / Malvaceae	—	ES826553
Raphanus sativus	Brassicales / Brassicaceae	—	EW738223
Arabidopsis thaliana	Brassicales / Brassicaceae	—	CB254580
Brassica napus	Brassicales / Brassicaceae	—	CX190866
Malus domestica	Rosales / Rosaceae	—	CN848744
Prunus persica	Rosales / Rosaceae	—	DY636044
Medicargo truncatula	Fabales / Leguminosae	—	TC96112
Glycine max	Fabales / Fabaceae	—	BE658784
Arachis hypogaea	Fabales / Fabaceae	—	CD038614

Populus trichocarpa	Malpighiales / Salicaceae	—	DT488487
Euphorbia esula	Malpighiales / Euphorbiaceae	—	DV153043
Manihot esculenta	Geraniales / Geraniaceae	—	DR087556
Vitis vinifera	Vitales / Vitaceae	—	EE063953
Persea americana	Laurales / Lauraceae	—	FD504330
Liriodendron tulipifera	Magnoliales / Magnoliaceae	—	CV004837

[a] Samples used in RT-PCR analysis.

[b] Accessions in bold are IRRI-IRGC accession No. and other accessions were collected by the authors.

[c] Sequences in bold were generated in this study and all the remaining sequences were downloaded from GenBank.
