# Supplementary figures and images for: Molecular evolution of the duplicated TFIIAγ genes in Oryzeae and its relatives
Source: BMC Evol Biol. 2010 May 4;10:128. doi: 10.1186/1471-2148-10-128 (PMC2887407; doi:10.1186/1471-2148-10-128)

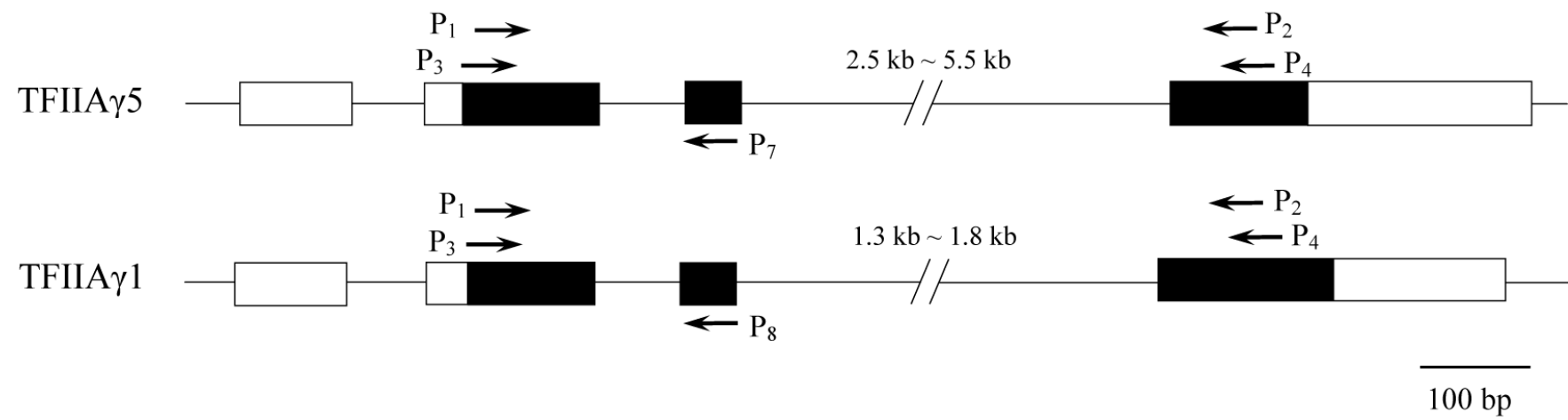

Supplement: Additional file 2 — Gene structure and the location of primers. Universal forward (P1 and P3) and reverse (P2 and P4) primers are shown above the genes and the copy-specific internal sequencing primers (P7 and P8) are shown below the gene. Exons are shown in boxes and the shaded boxes are coding regions. [file 1471-2148-10-128-S2.PDF]

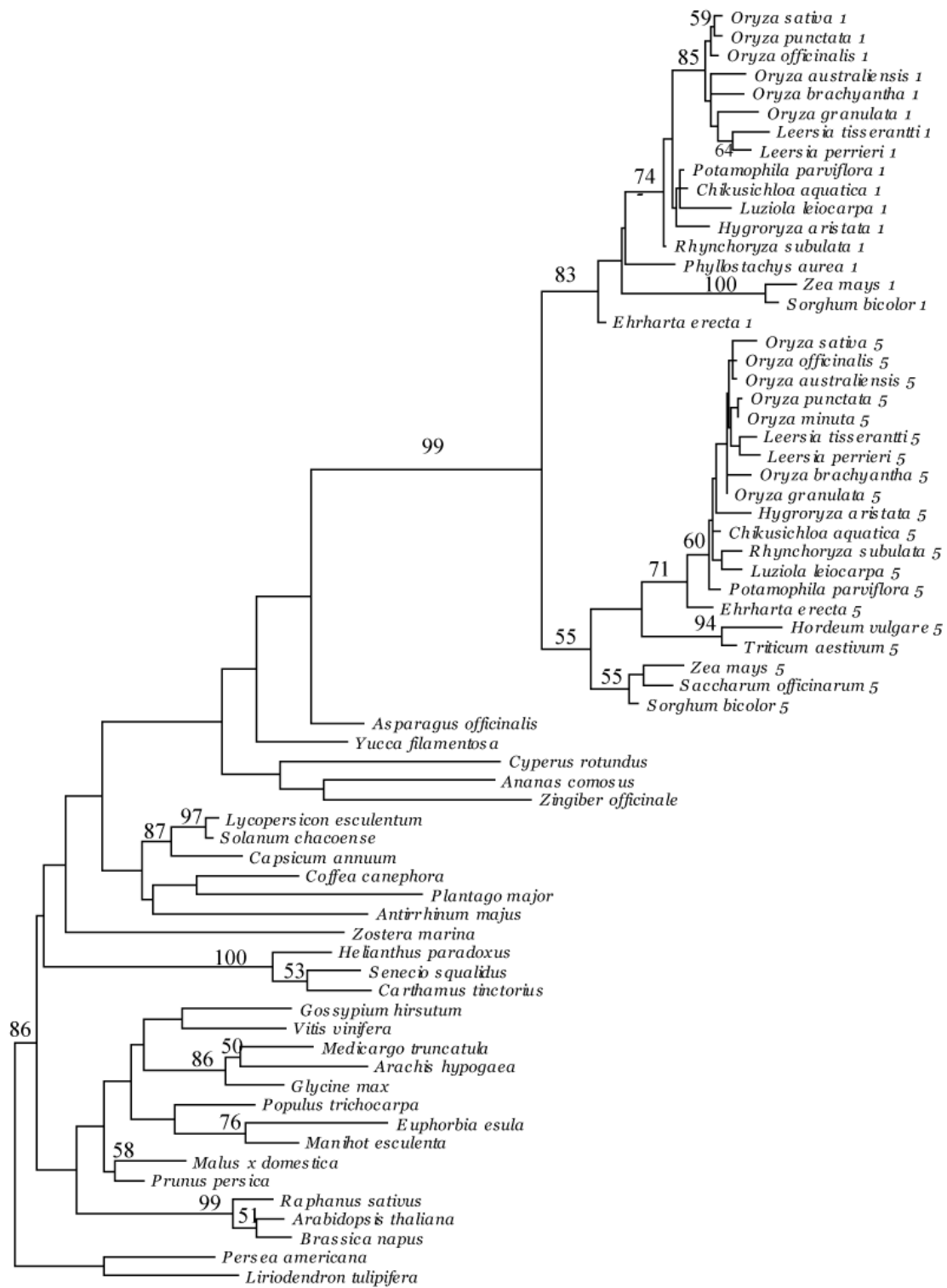

Supplement: Additional file 4 — Maximum likelihood tree using GTR+I +G model of evolution. Bootstrap values > 50% are shown above branches. [file 1471-2148-10-128-S4.PDF]
