# Supplementary material for: Molecular evolution of the duplicated TFIIAγ genes in Oryzeae and its relatives
Source: BMC Evol Biol. 2010 May 4;10:128. doi: 10.1186/1471-2148-10-128 (PMC2887407; doi:10.1186/1471-2148-10-128)
Supplement: Additional file 3 — GC contents (%) and ENC of TFIIAγ1 and TFIIAγ5 in Oryzeae species and its relative. [file 1471-2148-10-128-S3.PDF]

GC contents (%) and ENC of *TFIIAγ1* and *TFIIAγ5* in Oryzeae species and its relative

Species	GC_{total}		GC_1		GC_2		GC_3		ENC	
	GC_γ1	GC_γ5	GC1_γ1	GC1_γ5	GC2_γ1	GC2_γ5	GC3_γ1	GC3_γ5	ENC_γ1	ENC_γ5
Oryza sativa	52.7	51.3	46.3	46.3	34.8	35.8	76.8	71.6	53.2	40.0
Oryza punctata	52.6	50.5	46.3	47.3	34.8	35.8	76.8	68.4	48.2	41.8
Oryza officinalis	52.6	50.6	46.3	47.3	35.8	34.8	75.8	69.5	49.3	42.2
Oryza australiensis	51.9	50.5	45.2	47.3	35.8	34.8	74.7	69.4	49.6	41.5
Oryza brachyantha	53.4	51.2	46.3	47.3	35.8	34.8	77.9	71.5	42.7	43.7
Oryza granulata	52.0	50.5	46.3	47.3	34.7	34.8	74.8	69.4	49.7	42.0
Leersia perrieri	51.9	49.5	48.4	46.3	35.8	34.8	71.5	67.4	56.1	44.6
Leersia tisserantti	50.6	49.1	46.3	47.3	34.7	34.8	70.5	65.2	51.8	46.5
Potamophila parviflora	53.0	51.3	47.3	47.3	34.8	34.8	76.8	71.6	47.5	42.3
Chikusichloa aquatica	52.7	51.3	46.3	47.3	35.8	33.7	75.8	72.7	44.1	41.4
Rhynchoryza subulata	53.7	50.5	48.4	47.4	35.8	35.8	76.9	68.4	54.3	48.8
Hygroryza aristata	53.0	51.6	46.3	46.3	35.8	34.8	76.9	73.7	49.7	40.3
Luziola leiocarpa	53.3	50.9	49.5	46.3	34.8	34.8	75.8	71.5	46.5	44.6
Ehrharta erecta	55.4	51.2	49.4	47.4	35.8	34.7	81.0	71.6	35.9	40.4
Average	52.8	50.7	47.0	47.0	35.4	34.9	75.9	70.1	48.5	42.9
P-value (Pair-wise T test)	0.0 **		0.972		0.092		0.0 **		0.001**	

Pair-wise T test was performed between the corresponding GC_{total}, GC_1, GC_2, GC_3 and ENC of TFIIAγ1 and TFIIAγ5 copies in each species.

*, significant at $P < 0.05$ level; **, significant at $P < 0.01$ level.
