# Supplementary material for: Molecular evolution of the duplicated TFIIAγ genes in Oryzeae and its relatives
Source: BMC Evol Biol. 2010 May 4;10:128. doi: 10.1186/1471-2148-10-128 (PMC2887407; doi:10.1186/1471-2148-10-128)
Supplement: Additional file 6 — EST hits of grass TFIIAγ genes in GenBank EST database. [file 1471-2148-10-128-S6.PDF]

EST hits of grass *TFIIAγ* genes in GenBank EST database

TFIIAγ1			
Species	GenBank acc.	No. total hits for each species	Library type
Oryza sativa	CB097192	4	Drought Stress Panicle Library
	BI798012		10 days after anthesis
	CR292580		Unknown
	CR292640		Unknown
Sorghum bicolor	CF771824	4	Leaf library Drought-stressed before flowering
	CF771823		Leaf library Drought-stressed before flowering
	CF483781		Pollen
	CF483704		Pollen
Zea mays	BI180350	13	Immature leaf primordium and vegetative meristem
	BI233597		Immature leaf primordium and vegetative meristem
	BQ485252		Mature pollen
	EY963282		Ovary
	CO454127		Endosperm cDNA Library
	EC886352		Multiple tissues: 7 day seedling, 7 day seedling with 2 treatment, 4 hour UV-B treatment, 2 day osmotic stress, 7 day etiolated seedling and Juvenile leaf
	EC885724		Multiple tissues: 7 day seedling, 7 day seedling with 2 treatment, 4 hour UV-B treatment, 2 day osmotic stress, 7 day etiolated seedling and Juvenile leaf
	BM350890		Mixed Germinated seed and seedlings (1, 2, 8, 11 DAG), Mixed mature tissues (17, 21, 38, 69, 77 DAG), Kernels (3,5, 10, 15, 20, 25, 30, DAP), Adventious

			roots (65 DAG),Tassel (3-39 cm, 53 and 56 DAG), Immature ear (0.2-3.0 cm, 53, 56, 59 DAG), Husk (73 DAG), Silk, unpollinated first ear, ear shank, etiolated seedlings, callus, Cycloheximide-treated callus, Anaerobic treated seedlings, NAA (a-Naphthalene acetic acid)-treated seedlings, Kinetin-treated seedlings, ACPC(1-aminocyclopropane-1-carboxylix acid)-treated seedlings, Brassinolide-treated seedlings, ABA (Abscisic acid)-treated seedlings, GA (Gibberellic acid)-treated seedlings, JA (Jasmonic acid)-treated seedlings
	EB707035		Mixed (silks, husks, ears, pollen, shoot tips, leaf, root tips, whole seed, embryo)
	EB705434		Mixed (silks, husks, ears, pollen, shoot tips, leaf, root tips, whole seed, embryo)
	DV522145		Mixed (silks, husks, ears, pollen, shoot tips, leaf, root tips, whole seed, embryo)
	DY399012		Unknown
	CA826708		Unknown

TFIIAγ5

Species	GenBank acc.	No. total hits for each species	Library type
Oryza sativa	BI305732	50	Drought stress (leaf)
	BI305762		Drought stress (leaf)
	CB966887		Drought stress (leaf)
	AU057468		Mature leaf
	CI700382		4.5 leaf-stage leaf
	CI479845		4.5 leaf-stage leaf
	AU094261		Panicle at flowering stage
	C73323		Panicle at flowering stage

C98076	Callus
CI041307	Callus
D15390	Callus
CA753920	Root
CF281623	Rice etiolated leaf plasmid cDNA library
CF281624	Rice etiolated leaf plasmid cDNA library
CF281941	Rice etiolated leaf plasmid cDNA library
CF297694	Rice leaf plasmid cDNA library
CF293359	Rice leaf plasmid cDNA library
CF295167	Rice leaf plasmid cDNA library
CF296578	Rice leaf plasmid cDNA library
CF296579	Rice leaf plasmid cDNA library
CF296874	Rice leaf plasmid cDNA library
CF297693	Rice leaf plasmid cDNA library
CI689472	2nd week immature panicle
CI699832	2nd week immature panicle
CI459398	3rd week immature panicle
CI465713	3rd week immature panicle
CI472310	3rd week immature panicle
CI681147	3rd week immature panicle
CI686866	3rd week immature panicle
CI693017	3rd week immature panicle
AU031386	Immature leaf including apical meristem
CF333115	AtJMT-overexpressing transgenic rice plasmid cDNA library
CF333116	AtJMT-overexpressing transgenic rice plasmid cDNA library
CF986183	Tillering whole plant cDNA library
CF991338	Tillering whole plant cDNA library
CI026109	Etiolated shoot

	CI294906		Etiolated shoot
	CI312535		Shoot
	CI564074		Shoot
	CI583446		Shoot
	D40126		Shoot
	CI586304		Flower
	CI596913		Flower
	CI701719		25 days after pollination pistil
	CX103468		Whole plant
	CI674624		Unknown
	CR284051		Unknown
	CR284143		Unknown
	CT861692		Unknown
	CI699818		Unknown
Zea mays	BE638614	45	Tassel primordium
	BE638738		Tassel primordium
	BE639098		Tassel primordium
	AW267622		Tassel length from 0.1 to 2.5 cm
	BE186749		Tassels just after the transition from vegetative to inflorescence development
	BE224790		Tassels just after the transition from vegetative to inflorescence development
	BE510016		Tassels just after the transition from vegetative to inflorescence development
	DN203842		Vegetative Shoot Apical Meristem (SAM) and leaf primordia
	DN225306		Vegetative Shoot Apical Meristem (SAM) and leaf primordia
	DN227390		Vegetative Shoot Apical Meristem (SAM) and leaf primordia
	BG836506		2.5 cm of top of unfertilized ear Develop. stage: 6-7 days post-silk emergence
	BG836616		Developing kernels (sibcrossed) Develop. stage: 10-11 days post-silk emergence
	AI438550		Leaf primordia

DW530625	Egg cell
AW062024	Embryo
CV071969	Embryo sac
CD438730	Endosperm of 7-23DAP
CD437309	Endosperm of 7-23DAP
CD436174	Endosperm of 7-23DAP
DN559214	Embryo 7 days after pollination
DN559215	Embryo 7 days after pollination
CK367997	Root
CK827627	Root
CF634764	Root
CK370748	Root
CF060884	Seedling minus kernel
BG841882	Seedling and silk
BG842574	Seedling and silk
CF034253	Seedling minus kernel
DN213524	Shoot apex
BM335121	Mixed
BM334778	Mixed
BM268867	Mixed
CB350656	Mixed
BM349674	Mixed
BM074885	Mixed
DR802532	Mixed (silks, husks, ears, pollen, shoot tips, leaf, root tips, whole seed, embryo)
DR805033	Mixed (silks, husks, ears, pollen, shoot tips, leaf, root tips, whole seed, embryo)
DR970359	Mixed (silks, husks, ears, pollen, shoot tips, leaf, root tips, whole seed, embryo)
DV516037	Mixed (silks, husks, ears, pollen, shoot tips, leaf, root tips, whole seed, embryo)
EC858786	Mixed tissues (leaf, stem, floral bud) Develop. stage: twelve-leaf-old plants

			grown under normal conditions and drought-, salt-, and alkali co-stress for 48h
	EE037482		Mixed (7 day seedling, 7 day seedling with 2 day salt treatment, 4 hour 45°C heat shock, 24 hour 10°C treatment, 4 hour UV-B treatment, 2 day osmotic stress, 7 day etiolated seedling and Juvenile leaf.)
	EE037483		Mixed (7 day seedling, 7 day seedling with 2 day salt treatment, 4 hour 45°C heat shock, 24 hour 10°C treatment, 4 hour UV-B treatment, 2 day osmotic stress, 7 day etiolated seedling and Juvenile leaf.)
	DV514073		Mixed (silks, husks, ears, pollen, shoot tips, leaf, root tips, whole seed, embryo)
	DT944856		Mixed (silks, husks, ears, pollen, shoot tips, leaf, root tips, whole seed, embryo)
Triticum aestivum	CD866299	28	Root
	CD866300		Root
	CJ782541		Root
	CJ794391		Root
	CJ807847		Root
	CJ864544		Root
	CK193558		Root
	BE405810		Root Develop. stage: Five day old etiolated seedling root
	CA724752		Seedling
	BE471257		Drought-stressed seedling
	BJ223596		Crown of seedling
	BJ228321		Crown of seedling
	CK214190		Crown and leaf
	CJ673632		Seed DPA5
	BJ290238		Seed DPA30
	BJ296576		Seed DPA30
	CD897117		Grain (174 degrees per day after pollination)

	CJ641406		Dormant seed with cold treatment after water absorption
	BQ903064		Ta03_AAFC_ECORC_Fusarium_graminearum_inoculated_wheat_heads at anthesis stage
	CN008079		Wheat Fusarium graminearum infected spike
	CA594106		Anthers
	CA484144		Meiotic stages pre-meiosis-metaphase I Anther
	CA717203		Kernel
	CA708404		Kernel, 7 days after anthesis
	CA709799		Kernel, 7 days after anthesis
	CJ664995		Shoot grown under continuous light
	CJ697248		Shoot grown with cold treatment
	CJ591184		Shoot grown with cold treatment
Saccharum officinarum	CA094132	27	Pool of sugarcane calli submitted to low (4°C) and high (37°C) temperature stress
	CA106969		Seedlings inoculated with Herbaspirilum rubrisubalbicans
	CA196811		Seedlings inoculated with Gluconacetobacter diazotroficans
	CA113293		Lateral buds from field grown adult plants
	CA266558		Lateral buds from plants adult plants growing in greenhouse
	CA119459		Leaf roll from field grown adult plants
	CA123720		Leaf roll from field grown adult plants
	CA184744		Fourth apical stalk internodes of adult plants
	CA186566		Fourth apical stalk internodes of adult plants
	CA248613		Inflorescence at beginning of development (1cm-long)
	CA252259		Inflorescence at beginning of development (1cm-long)
	CA229688		Base of developing inflorescence (5cm-long)
	CA291689		Developing inflorescence and rachis (10cm-long)
	CA299350		Developing inflorescence and rachis (10cm-long)

	CA245878		Developed inflorescence (20cm-long) without rachis
	CO373849		Roots Develop. stage: 3 months post-planting
	CA260612		Root apex from adult plants
	CA283844		Developing seeds
	CF574237		Internodes 6-11 Develop. stage: 12 months post-planting
	CA094322		Pool of sugarcane calli submitted to low (4°C) and high (37°C) temperature stress
	CA188938		Pool of sugarcane calli submitted to low (4°C) and high (37°C) temperature stress
	CA074241		Apical meristem and tissues surrounding of mature plants
	CA186639		Fourth apical stalk internodes of adult plants
	CA107034		Seedlings inoculated with Herbaspirilum rubrisubalbicans
	CA109873		Seedlings inoculated with Herbaspirilum rubrisubalbicans
	CA109955		Seedlings inoculated with Herbaspirilum rubrisubalbicans
	BQ534160		Leaves After floral induction
Hordeum vulgare	AJ475344	15	Callus
	AV916338		Shoots
	AV921769		Shoots
	BG417871		Testa/pericarp
	CA022917		Pericarp
	CA029039		Pericarp
	BI950776		Spike
	BJ480181		Heading stage top three leaves
	CV053747		Endosperm
	CX629985		Roots
	CX630179		Roots

	BI779255		Root, 3 week, hydroponic grown, no treatment
	BJ484771		Heading stage top three leaves
	FD527116		Pericarp 0-7 DAP (days after pollination)
	FD527589		Pericarp 0-7 DAP (days after pollination)
Sorghum bicolor	CN152422	4	Wounded leaves
	CN152492		Wounded leaves
	CX622784		GA- or brassinolide-treated seedlings
	CX622871		GA- or brassinolide-treated seedlings
Eragrostis curvula	EH185773	1	Panicles
Aegilops speltoides	CD491723	1	Premeiotic anthers
Oryza minuta	CB210904	1	Immature leaf
Triticum monococcum	BG314347	1	Early reproductive apex
