# Supplementary material for: Molecular evolution of the duplicated TFIIAγ genes in Oryzeae and its relatives
Source: BMC Evol Biol. 2010 May 4;10:128. doi: 10.1186/1471-2148-10-128 (PMC2887407; doi:10.1186/1471-2148-10-128)
Supplement: Additional file 5 — Amino acid alignment of the TFIIAγ genes. 2D structure in the bottom is predicted by PredictProtein http://www.predictprotein.org/ using O. sativa sequences as references. H represents the alpha helix and E the beta strand. [file 1471-2148-10-128-S5.PDF]

```
                                     10        20        30        40        50        60        70        80        90
                            ....|....|....|....|....|....|....|....|....|....|....|....|....|....|....|....|....|....|....|
Oryza sativa_1              MATFELYRRSTIGMCLTDTLDDMVSSGALSPELAIQVLVQFDKSMTSALEHQVKSKVTVKGHLHTYRFCDNVWTFILTDAIFKNEEITETINKVK
Oryza punctata_1            ------.........................................................................P.............--
Oryza officinalis_1         ------..........................................................................T............--
Oryza australiensis_1       ------.......T.............T.C...............................................K..T............--
Oryza brachyantha_1         ------.......................................................................R..T.....M......--
Oryza granulata_1           ------...................................................I...................K..T..........SN--
Leersia tisserantti_1       ------.....................G.................................................K..T..........G.--
Leersia perrieri_1          ------.....................G.................................................K..T............--
Potamophila parviflora_1    ------...........E............................N..........S......................T......A.....--
Chikusichloa aquatica_1     ------...........E............................N..........S......................T......A...S.--
Rhynchoryza subulata_1      ------...........E............................N..........S......................T.........VG.--
Luziola leiocarpa_1         ------...........E...E..L..T...D..............D.................................T......A...G.--
Hygroryza aristata_1        ------...........E............................N..........S......................T..........S.--
Ehrharta erecta_1           ------...........E............................D...N.............................T.........VG.--
Phyllostachys aurea_1       ------.......L...E.........T..................D...N......S......................P......S..VS.--
Zea mays_1                  .............T...E...EL.....V.................E...M......S......................T..S...Q..LGR..
Sorghum bicolor_1           .............T...E...EL.....V.................E...M......N......................T..S...Q..LG...
2D structure                --EEEEE----HHHHHHHHHHHHHH-----HHHHHHHHHHHHHHHHHHHHHH----EEEEEEEEEEEE---EEEEEEE-EEEEE---EEE-----
```

```
                                     10        20        30        40        50        60        70        80        90
                            ....|....|....|....|....|....|....|....|....|....|....|....|....|....|....|....|....|....|
Oryza sativa_5              MATFELYRRSTIGMCLTETLDEMVSSGTLSPELAIQVLVQFDKSMTEALENQVKSKVSIKGHLHTYRFCDNVWTFILTEASFKNEETTEQVGKVK
Oryza punctata_5            ------..........................................................................P....D.......--
Oryza officinalis_5         ------..........................................................................Q............--
Oryza australiensis_5       ------..........................................................................Q....D.......--
Oryza brachyantha_5         ------..........................................................................Q............--
Oryza granulata_5           ------..........................................................................Q............--
Leersia perrieri_5          ------..........................................................................Q............--
Leersia tisserantti_5       ------..........................................................................Q............--
Potamophila parviflora_5    ------..........................................................................Q............--
Chikusichloa aquatica_5     ------..........................................................................Q.....N.D....--
Rhynchoryza subulata_5      ------..........................................................................A............--
Luziola leiocarpa_5         ------..........................................................................Q............--
Hygroryza aristata_5        ------..........................................................................N............--
Ehrharta erecta_5           ------...................T.........................E.....T....................D.Q............--
Hordeum vulgare_5           .........................................................TV...................D.Q.....I....S...
Triticum aestivum_5         ..............................................D...T......TV...................D.Q..............
Zea mays_5                  .........................N....................D..........TV...................D.......A........
Sorghum bicolor_5           ..............................................D..........N....................D................
Saccharum officinarum_5     ........................NN............I.......D..........N.....................................
2D structure                --EEEEEE---HHHHHHHHHHHHHH-----HHHHHHHHHHHHHHHHHHHHHH----EEEEEEEEEEEEE--EEEEEEEEEEEEE---EEEE----
```
